# Land-Use Change Enhanced SOC Mineralization but Did Not Significantly Affect Its Storage in the Surface Layer

**DOI:** 10.3390/ijerph19053020

**Published:** 2022-03-04

**Authors:** Haikuo Zhang, Xuli Zheng, Yanjiang Cai, Scott X. Chang

**Affiliations:** 1 State Key Laboratory of Subtropical Silviculture, Zhejiang A&F University, Hangzhou 311300, China; hkzhang@zafu.edu.cn (H.Z.); scott.chang@ualberta.ca (S.X.C.); 2 College of Environmental and Resource Sciences, Zhejiang A&F University, Hangzhou 311300, China; 3 Anji County Lingfeng Temple Forest Farm, Huzhou 313302, China; zxl0326@163.com; 4 Department of Renewable Resources, University of Alberta, 442 Earth Sciences Building, Edmonton, AB T6G 2E3, Canada

**Keywords:** carbon-degrading enzyme activity, forest conversion, soil organic carbon mineralization

## Abstract

To achieve carbon (C) neutrality and mitigate climate change, it is crucial to understand how converting natural forests to agricultural plantations influences soil organic C (SOC) mineralization. In this study, we investigated the impact of converting evergreen broadleaf forests (EBF) to extensively managed Moso bamboo (*Phyllostachys edulis* (Carriere) J. Houzeau) plantations (MBP) in subtropical China on SOC mineralization rate; the concentrations of labile SOC fractions such as dissolved organic C (DOC), microbial biomass C (MBC), and readily oxidizable C (ROC); the activities of C-degrading enzymes (cellobiohydrolase and phenol oxidase); and the abundance of C-degrading enzyme-encoding genes (*cbh*I and *lcc*). Three paired soil samples were taken from the surface layer (0–20 cm) of adjacent EBF-MBP sites in Anji County, Zhejiang province. Results showed that converting EBF to MBP significantly increased the SOC mineralization rate as well as soil pH, MBC, cellobiohydrolase, and phenol oxidase activities, and *cbh*I gene abundance, but did not change other soil properties described above. In addition, structural equation modelling (SEM) showed that the conversion increased SOC mineralization rate through increasing soil pH, *cbh*I gene abundance, MBC, and cellobiohydrolase and phenol oxidase activities. Our novel finding that converting EBF to extensively managed MBP enhanced SOC mineralization via increasing the activities of C-degrading enzymes suggests that C-degrading enzymes were a key factor regulating SOC mineralization in the extensively managed subtropical bamboo plantations.

## 1. Introduction

Globally, approximately 39% of soil organic carbon (SOC) is stored in forest ecosystems [1]. In forest ecosystems, SOC accounts for about 67% of the total carbon (C) pool [2]. Therefore, the mineralization of forest SOC plays a vital role in global C neutrality and atmospheric carbon dioxide (CO_2_) concentration [3,4]. One of the critical factors affecting forest SOC sequestration is the changes in land use. Conversion of natural forests to more intensively managed forests or agricultural lands has frequently been done in the past several decades across the world to obtain more economic benefits and meet the demand for food and fiber due to increasing population [5,6]. For example, 83% of new agricultural lands in the tropics was converted from natural forests between 1980 and 1990 [7]. Generally, forest conversion and field management practices that follow the conversion change above- and below-ground litter input to soils [8,9], which will inevitably affect the dynamics of SOC [3,10]. The conversion of natural forests to agroforestry plantations [3,11] and pine plantations [12] has been found to increase CO_2_ emissions. However, some studies of converting natural forests to forest plantations found negative or no effect on soil CO_2_ emissions [13,14,15]. Therefore, the mechanisms mediating soil CO_2_ emissions under forest conversion need to be further studied.

The mineralization of SOC is one of the main sources of soil CO_2_ emissions [16,17]; the rate of SOC mineralization thus reflects the dynamics of SOC [18,19]. In general, environmental factors such as humidity and temperature are thought to influence the SOC mineralization [1,4]. Additionally, the labile SOC fractions such as readily oxidizable C (ROC) and dissolved organic C (DOC) generally serve as the main C sources for microbial respiration that controls SOC mineralization [20,21]. Due to its high sensitivity to environmental changes, labile SOC fractions are generally considered a potential predictor of SOC mineralization after forest conversion [22,23,24,25]. For example, Lin et al. [13] found that converting natural evergreen broadleaf forests to intensively managed Moso bamboo (*Phyllostachys edulis* (Carriere) J. Houzeau) plantations decreased SOC mineralization rate, which was significantly related to soil ROC and DOC. Lyu et al. [10] also observed that cumulative SOC mineralization in the topsoil was significantly decreased by forest conversion due to the depletion of soil DOC and microbial biomass carbon (MBC). In addition to the availability of labile SOC, extracellular enzymes secreted by soil microbes play an essential role in SOC mineralization, as they catalyze the breakdown of soil organic matter (SOM) until the products can be assimilated by microbes [26,27]. Cellulose and lignin are two of the most abundant organic compounds in SOM and the main energy source of microbe-mediated SOC mineralization [28,29,30]. For example, cellobiohydrolase can hydrolyze cellulose, and phenol oxidase can oxidize lignin; these two C-degrading enzymes are crucial for SOC mineralization [30,31,32]. Soil C-degrading enzyme activities can be also affected by forest conversion. For instance, Lin et al. [13] observed that forest conversion significantly decreased soil cellobiohydrolase activity. Zhang et al. [33] found that converting forests into croplands significantly increased the activity of soil phenol oxidase in the subtropics. Moreover, soil enzyme activities were closely related to associated enzyme-encoding genes [34,35]. Soil cellobiohydrolase activity was found to be positively correlated with the abundance of fungal *cbh*I gene in a subtropic region [36]. In spite of this, few studies about forest conversion examined both soil C-degrading enzyme activities and the associated enzyme-encoding gene abundances. In addition, there is also a lack of studies on how soil C-degrading enzymes and associated enzyme-encoding genes mediate SOC mineralization in the context of forest conversion.

Moso bamboo plantations are one of the most important forest resources in subtropical China. Most Moso bamboo plantations were converted from natural forests due to high economic benefits provided by the Moso bamboo plantations in the past several decades [37,38]. Previous studies have reported the effects of converting natural forests to intensively managed Moso bamboo plantations on SOC dynamics [13,37,39,40]. However, extensively managed Moso bamboo plantations converted from natural forests are also common in subtropical China [41]. Unlike intensively managed Moso bamboo plantations, extensively managed Moso bamboo plantations are generally not managed with deep ploughing, fertilizer application, or understory vegetation removal [41]. The characteristics of SOC mineralization after forest conversion to extensively managed Moso bamboo plantations may therefore be different with those in the intensively managed Moso bamboo plantations. It is essential to explore the characteristics of SOC mineralization after converting natural forests to extensively managed Moso bamboo plantations.

The present study aimed to investigate the effect of converting natural forests to extensively managed Moso bamboo plantations on SOC mineralization and the relationship between SOC mineralization and labile SOC fractions (DOC, ROC, and MBC), C-degrading enzyme (cellobiohydrolase, phenol oxidase) activities, and the abundance of associated enzyme-encoding genes (*cbh*I and *lcc*). We hypothesize that: (1) converting natural forests to extensively managed Moso bamboo plantations will not decrease the concentrations of total SOC and labile SOC fractions, as the aboveground vegetation was preserved in extensively managed MBP; (2) such forest conversion mediates SOC mineralization rates by regulating both the C-degrading enzyme activities and labile SOC fractions because SOC mineralization and the C-degrading enzyme activities and the labile SOC fractions are closely linked; and (3) the change in the activities of the C-degrading enzymes influenced by such forest conversion is linked to the abundance of associated enzyme-encoding genes.

## 2. Materials and Methods

### 2.1. Study Site and Soil Sampling

The study was conducted in a typical hilly region in Anji County (30°41′ N, 119°46′ E), in the northern part of Zhejiang Province, China. The study area has a north subtropical monsoon climate, with a mean annual temperature of 17.0 °C and a mean annual precipitation of 1706.2 mm. The soil is classified as a Ferralsol [42]. The native vegetation in the study area is the natural evergreen broadleaf forest (EBF). The EBF has existed for at least 100 years, and the tree species were dominated by *Cyclobalanopsis glauca*, *Castanopsis sclerophylla*, and *Schima superba Gardn*. Local farmers have been converting part of the EBF into the Moso bamboo plantation (MBP) since 2000 with the increasing demand for bamboo resources. The MBP was extensively managed after the conversion, including harvesting of bamboo stems and shoots (without deep ploughing, fertilizer application, and understory vegetation removal). Three pairs of sampling plots (two different land-use types in each pair, i.e., natural evergreen broadleaf forest and Moso bamboo plantation) were selected after a field survey in June 2019. All sampling plots had the same land-use change history and the similar geographical characteristic (altitude, slope, aspect, soil parent materials, and so on). Each sampling plot was approximately 20 × 20 m.

We collected surface (0–20 cm) soil samples two times in the growing season (July and October 2019). Briefly, five soil cores were obtained from the center and four corners of each sampling plot, mixed to form a composite sample per plot, and brought back to the laboratory. In the laboratory, soil samples were passed through a 2 mm sieve to remove visible roots and divided into three parts, i.e., air-dried at room temperature, stored at 4 °C, and stored at −80 °C for subsequent analysis.

### 2.2. Determination of Soil Physicochemical Properties and Labile SOC Fractions

Soil bulk density (BD) was measured by the bulk density ring method in July 2019. Soil texture was characterized using a laser size detector (Malvern Panalytical Mastersizer 3000E, UK). Soil pH was determined in a suspension with a 1:2.5 (*w*:*v*) soil to distilled water ratio using a pH meter (Mettler Toledo Seven Compact S210, Zurich, Switzerland). Soil moisture content was determined by oven-drying soil samples at 105 °C to a constant mass. The concentration of SOC was determined by the oxidation method using K_2_Cr_2_O_7_-H_2_SO_4_ [43]. The total N concentration was determined by an auto-analyzer (Hanon K9860, Shandong, China) after micro-Kjeldahl digestion [44]. The following formula was used to calculate the SOC storage: SOC storage (Mg ha^−1^) = SOC (Kg Mg^−1^) × BD (Mg m^−3^) × depth (m) × 10.

To determine the concentration of DOC, 5 g (on an oven-dry basis) fresh soil was extracted in 25 mL deionized water, and the slurry was shaken at 250 rpm for 30 min on a shaker. The sample was then centrifuged at 5000× *g* for 20 min and filtered through a 0.45-μm membrane filter (JINTENG Φ25, Tianjin, China). The C concentration in the extracts were determined using a TOC analyzer (Multi N/C3100, Analytik Jena AG, Jena, Germany). The concentration of MBC was analyzed using the chloroform fumigation-extraction method [45,46]. Briefly, two 5 g (on an oven-dry basis) fresh soil samples (one fumigated and one non-fumigated) were extracted in 25 mL 0.5 M K_2_SO_4_. The extracts were filtered through 0.45-μm membrane filters and then the soluble C concentration was measured by the TOC analyzer. The concentration of MBC was calculated as the difference in C concentration in the extracts between fumigated and non-fumigated soils, and based on an extraction efficiency factor of 0.45 [47]. The ROC concentration was measured by the KMnO_4_ oxidation method [48]. Briefly, 2 g air-dried soil sample was extracted in 25 mL 0.333 M K_2_MnO_4_ solution and shaken at 200 rpm for 1 h, then centrifuged at 5000× *g* for 5 min. The supernatant was passed through qualitative filter papers (Whatman^®^ No. 42, Cytiva, Buckinghamshire, UK), and then the absorbance was determined at 565 nm using a UV spectrophotometer (UV-2600, Shimadzu, Kyoto, Japan).

### 2.3. Determination of Soil C-Degrading Enzyme Activities

We analyzed the activities of cellobiohydrolase and phenol oxidase using fluorometric techniques with 96-well microplates [49,50]. Briefly, 1.5 g (on an oven-dry basis) fresh soil was weighed and suspended in 125 mL sodium acetate buffer (50 mM, pH = 5.0). For cellobiohydrolase, the three rows of the sample group in the 96-well microplate included 200 μL soil slurries combined with a 50 μL cellobiohydrolase substrate (200 μM), 200 μL soil slurries combined with 50 μL sodium acetate buffer, and 200 μL soil slurries combined with 50 μL standard substrate of 4-methylumbelliferone; the three rows of the control group included 200 μL sodium acetate buffer combined with 50 μL cellobiohydrolase substrate, 250 μL sodium acetate buffer, and 200 μL sodium acetate buffer combined with 50 μL 4-methylumbelliferone standard substrate. For phenol oxidase, the two rows of the sample group included 200 μL soil slurries combined with 50 μL L-DOPA (25 mM) and 200 μL soil slurries combined with 50 μL sodium acetate buffer; the two rows of the control group included 200 μL sodium acetate buffer combined with 50 μL L-DOPA, and 250 μL sodium acetate buffer. The microplate of cellobiohydrolase was incubated in the dark at 25 °C for 3 h, and phenol oxidase was incubated in the dark at 25 °C for 24 h. The fluorescence value of cellobiohydrolase with 365 nm excitation and 450 nm emission, and the absorbance value of phenol oxidase with 450 nm absorbance were determined using a microplate reader (Biotek^®^ Synergy H1, Winooski, VT, USA). The activities of cellobiohydrolase and phenol oxidase were expressed as nmol g^−1^ h^−1^ and μmol g^−1^ h^−1^, respectively.

### 2.4. Soil DNA Extraction and Quantitative PCR

The PowerSoil^®^ Pro DNA Isolation Kit (MOBIO, Qiagen, Germany) was used for soil DNA extractions. The ABI QuantStudio 3 Real Time PCR System (ABI QuantStudio 3, Thermo Fisher Scientific, Germany) was used to quantify the abundance of fungal C-degrading functional genes *cbh*I and *lcc* in the soils. The amplification primers of fungal *cbh*I and *lcc* are fungcbhIF (ACC AAY TGC TAY ACI RGY AA)/fungcbhIR (GCY TCC CAI ATR TCC ATC) [28] and fungCu1F (CAY TGG CAY GGN TTY TTY CA)/fungCu2R (RCT GTG GTA CCA GAA NGT NCC) [51], respectively. The reaction mixture system (20 μL) contained 10 μL SYBR^®^ Premix EX TaqTM (Takara RR420a, Shiga, Japan), 0.2 μL (50 mM) forward primer, 0.2 μL (50 mM) reverse primer, 1 μL template DNA (1–10 ng), and 8.6 μL ddH_2_O. The reaction conditions were: pre-denaturation at 95 °C for 5 min, followed by 35 cycles of denaturation at 95 °C for 30 s, annealing at 56 °C (*lcc*), or 57 °C (*cbh*I) for 30 s, extension at 72 °C for 30 s; the final extension was 15 min at 72 °C. The amplification efficiency of the genes was 89–105%, with R^2^ values more than 0.993. The target gene copy numbers were determined by a standard curve generated using purified template plasmid DNA.

### 2.5. Determination of Soil Organic C Mineralization Rates

A one-month aerobic incubation was conducted to determine the SOC mineralization rate. Briefly, 30 g (on an oven-dry basis) fresh soil of each sample was adjusted to 60% water holding capacity by adding deionized water and stored in a 250 mL flask at 25 °C incubator for 33 days. The flasks were weighed every 2–3 days, and deionized water was added as needed to keep soil moisture content constant. Headspace gas samples were collected from flasks on days 1, 2, 3, 5, 7, 9, 11, 16, 21, 27, and 33. Before each gas sampling, the flasks were sealed tightly using rubber stoppers and liquid silicone rubber (NQ-704 adhesive sealant, Nanjing, China). A 20 mL volume of air was injected into the flask with a syringe and mixed with the gas in the headspace; a 20 mL gas sample was then withdrawn to ensure the internal and external pressure balance of the flasks. The 20 mL gas sample collected was injected into a 12 mL vacuum vial (LabCo Exetainer^®^, High Wycombe, UK) and used as the background sample. The flasks were then placed into a 25 °C incubator for 4 h and another 20 mL sample of headspace gas was collected from the flask and injected into a 12 mL vacuum vial. The CO_2_ concentrations from all gas samples were determined using a gas chromatograph (Agilent^®^ 7890B, Santa Clara, CA, USA). The difference of the CO_2_ concentrations between the two sampling times was used to calculate the soil CO_2_ emission rate (C_R_) for the day. The cumulative total CO_2_-C emission (mg kg^−1^) during the 33-day incubation period was defined as the cumulative amount of SOC mineralization (mg kg^−1^). The following formula was used to calculate the cumulative amount of SOC mineralization (C_min_):

C_R_ = [(28/22.41) × ΔC × V × 10^−6^ × 237]/[M × Δt × 10^−3^ × (237 + T)] (1)

C_min_ = ∑(*i* = 1)^n (C_R(*i*)_ + C_R(*i*+1)_)/2 × (t*_i_*_+1_ − t*_i_*) × 24 (2)

where C_R_ is the soil CO_2_ emission rate (mg C kg^−1^ dry soil h^−1^); ΔC is the change in CO_2_ concentrations (ppmv) between incubation times of 0–4 h; V is the headspace volume (mL) of the flask; M is the dry weight of the soil (g); Δt is the incubation time (h); T is the incubation temperature (°C); C_min_ is the cumulative CO_2_-C emission (mg C kg^−1^ dry soil); and C_R(*i*)_ and C_R(*i*+1)_ are the CO_2_ emission rates at the time t*_i_* and t*_i_*_+1_, respectively.

The SOC mineralization rates (mg kg^−1^ day^−1^) were calculated as the average daily CO_2_ released during the 33-day incubation period.

### 2.6. Statistical Analyses

The data are presented as mean ± standard error (SE) (n = 3). After evaluating and ensuring that the distribution is normal and variance is homogeneous, paired *t*-tests were conducted to evaluate the treatment (land-use type and sampling time) on soil properties such as soil BD, pH, moisture content, SOC concentration, TN concentration, C/N, SOC storage, labile SOC concentration, enzyme activity, functional gene copy number, and SOC mineralization rate. The statistical analyses were conducted with SPSS 18.0 software (SPSS Inc., Chicago, IL, USA). All graphs were drawn using the “ggplot2” package in R software. Differences were considered significant at *p* < 0.05 in all analyses.

A structural equation model (SEM) was constructed using the “piecewiseSEM” package of R software to test hypothetical relationships. In our SEM, we only selected soil properties that were significantly affected by the forest conversion, including pH, MBC concentration, *cbh*I gene abundance, cellobiohydrolase activity, phenol oxidase activity, and SOC mineralization rate. The SEM is based on the assumption that forest conversion affects soil pH, MBC concentration, and *cbh*I gene abundance, which in turn affect C-degrading enzyme activities and the SOC mineralization rate. Additionally, the fitness of the SEM was checked by the Fisher’s C test and Akaike information criterion (AIC), and the path was revised according to the above criteria to achieve the best fit.

## 3. Results

### 3.1. Soil Physicochemical Properties and Labile SOC Fractions

**Figure 1:**
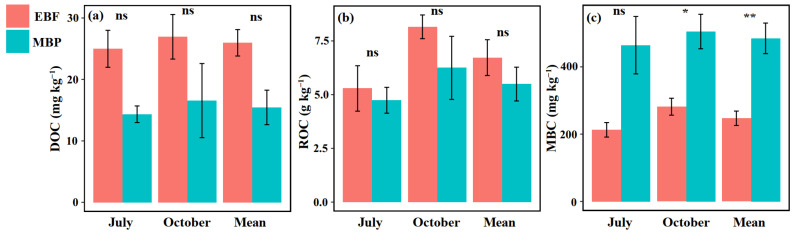
Effect of conversion of evergreen broadleaf forests (EBF) to extensively managed Moso bamboo plantations (MBP) on the concentration of (**a**) DOC, (**b**) ROC, and (**c**) MBC. * and ** indicate significant differences at the level of at *p* < 0.05 and *p* < 0.01, respectively (n = 3). The error bars in the graphs represent standard errors of the mean (SE).

Soils from the EBF and MBP were acidic, with soil pH ranging from 4.12 to 4.75. Regardless of the sampling time, the soil pH was always higher in MBP than in EBF (Table 1). Conversion of EBF to MBP did not significantly affect soil BD, texture, moisture content, SOC concentration and storage, TN concentration, C/N ratio (Table 1), DOC, or ROC (Figure 1a,b). Soil MBC concentration was significantly higher in MBP than in EBF at each sampling time (Figure 1c).

### 3.2. SOC Mineralization Rate, Soil Enzyme Activities and Functional Genes Abundance

**Figure 2:**
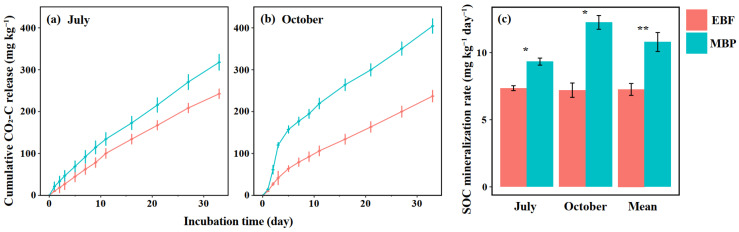
Effect of conversion of evergreen broadleaf forests (EBF) to extensively managed Moso bamboo plantations (MBP) on the cumulative CO_2_-C release in (**a**) July and (**b**) October, and (**c**) the SOC mineralization rate. * and ** indicate significant differences at the level of *p* < 0.05 and *p* < 0.01, respectively (n = 3). The error bars in the graphs represent standard errors of the mean (SE).

The SOC mineralization was very rapid at the beginning of the incubation but declined gradually with time for the two land-use types (Figure 2a,b), and the conversion of EBF to MBP all significantly increased SOC mineralization rate (Figure 2c). The overall mean SOC mineralization rate of the two sampling times in the MBP was 1.49 times higher than that in the EBF (*p* < 0.01, Figure 2c).

**Figure 3:**
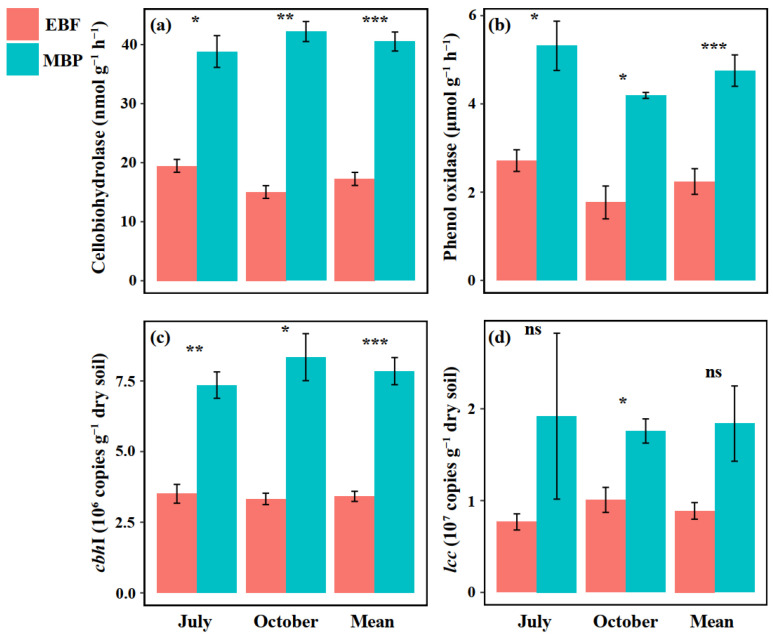
Effect of conversion of evergreen broadleaf forests (EBF) to extensively managed Moso bamboo plantations (MBP) on (**a**) cellobiohydrolase activity, (**b**) phenol oxidase activity, (**c**) *cbh*I gene abundance, and (**d**) *lcc* gene abundance. *, **, and *** indicate significant differences at the level of at *p* < 0.05, *p* < 0.01, and *p* < 0.001, respectively (n = 3). The error bars in the graphs represent standard errors of the mean (SE).

Regardless of sampling time, soil cellobiohydrolase and phenol oxidase activities and *cbh*I gene abundance were all significantly higher in MBP than in EBF (Figure 3a,c). Soil *lcc* gene abundance was significantly higher in MBP than in EBF only in October (Figure 3d).

### 3.3. Relationship between SOC Mineralization Rate and Soil Properties

**Figure 4:**
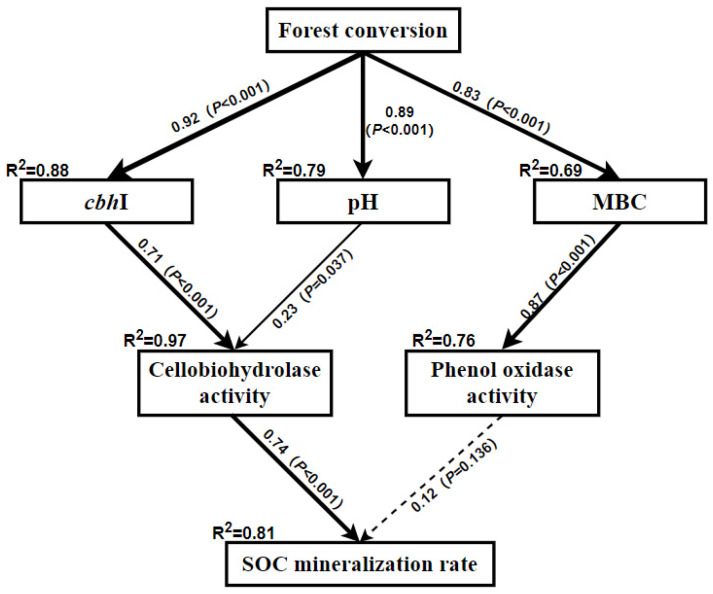
Structural equation modeling (SEM) of the effects of conversion of native broadleaf forests (EBF) to extensively managed Moso bamboo plantations (MBP) on SOC mineralization rates. Results of model fitting: *p* = 0.094; df = 26; AIC = 89.85; Fisher’s C = 49.85. The R^2^ values related with response variables mean the proportion of variation explained by relationships with other variables. The values related with arrows are standardized path coefficients. The solid arrows indicate significant effects (*p* < 0.05) and dotted arrows indicate no significant effects (*p* > 0.05).

Forest conversion directly increased soil pH (*p* < 0.001), MBC concentration (*p* < 0.001), and *cbh*I abundance (*p* < 0.001) (Figure 4). Furthermore, a large proportion of the variation of soil cellobiohydrolase activity was described by soil *cbh*I (*p* < 0.001) and pH (*p* = 0.037), and the phenol oxidase activity variation was mainly explained by soil MBC (*p* < 0.001) (Figure 4). In addition, most of the variation (74%) in the SOC mineralization rate was explained by soil cellobiohydrolase activity (*p* < 0.001) (Figure 4). The SEM showed that forest conversion increased soil pH and the abundance of *cbh*I gene, and the latter indirectly increased SOC mineralization rate through increasing the activity of cellobiohydrolase (Figure 4).

## 4. Discussion

Although many studies have reported the effect of forest conversion on SOC mineralization, they mainly focused on the conversion of natural forests into intensively managed agricultural lands [9,13]. How the conversion of natural forest into extensively managed bamboo plantations affects SOC mineralization is still poorly understood. Interestingly, we found that converting EBF into extensively managed MBP significantly increased SOC mineralization rate (Figure 1) but did not influence SOC storage (Table 1) and the concentration of labile SOC fractions, i.e., DOC and ROC (Figure 2) in the surface layer (0–20 cm). In spite of this, converting EBF to extensively managed MBP still increased the risk of C loss via enhanced SOC mineralization.

The increase in the cumulative SOC mineralization in our finding was consistent with Wang et al. [52], who found that the cumulative soil CO_2_-C emissions were significantly higher in the *Cunninghamia lanceolata* Hook and *Michelia macclurei* Dandy plantations than in the natural forest long after forest conversion. The significant correlation between labile SOC fractions and cumulative SOC mineralization was also in agreement with several previous studies [13,39,52]. Interestingly, our results showed that converting EBF to MBP only increased the concentration of MBC but did not change the other labile SOC fractions, i.e., DOC and ROC (Figure 2). In addition, our SEM also showed that there was no significant direct relationship between MBC and SOC mineralization (Figure 4). Therefore, we can exclude the possibility that forest conversion increased the SOC mineralization rate via changing soil labile SOC concentrations in the current study. Another possible reason for the enhanced SOC mineralization is the increased activities of C-degrading enzymes [30,32,53]. In the present study, converting EBF to MBP significantly increased the activities of cellobiohydrolase and phenol oxidase (Figure 3), which is likely to enhance cellulose and lignin degradation and SOC mineralization in the MBP soil. Short-term incubation experiments also demonstrated that the change in SOC mineralization rate after forest conversion was significantly affected by the activities of C-degrading enzymes such as cellobiohydrolase, β-glucosidase, and cellulase [13,52]. Our SEM analysis also showed that increasing the activities of C-degrading enzymes after forest conversion increased the SOC mineralization rate. However, it is worth noting that in the SEM the increased phenol oxidase activity following the forest conversion did not influence (*p* = 0.136) SOC mineralization rate (Figure 4), which could be due to the fact that phenol oxidase is only responsible for degrading recalcitrant organic compounds (e.g., lignin) into smaller ones during the SOC mineralization, whereas the key step of simple organic compound degradation is mainly accomplished by hydrolytic enzymes such as cellobiohydrolase [31,34,54]. Briefly, the increased cellobiohydrolase activity after forest conversion played a greater role than phenol oxidase in the mineralization of SOC. The cellobiohydrolase activity might be influenced by the increased soil pH and *cbh*I gene abundance after forest conversion in the present study. Soil pH can change the enzyme activity via impacting the stability of soil enzymes [55]. Zhang et al. [33] also found that the soil C-degrading enzyme activities were driven by soil pH after land-use change in a subtropical ecosystem. In this study, the increase of soil pH might not be caused by the changes in the nature of soil parent materials, because the land-use change did not significantly change the soil basic properties, such as bulk density, texture, or carbon and nitrogen concentration (Table 1). Many studies found that the fluctuations in soil pH could be attributed to the input of plant materials [56,57,58]. Therefore, the increase in soil pH was probably due to the changes in the organic matter input following the land-use change in the present study. Furthermore, Nannipieri et al. [34,59] indicated that the enzyme-encoding genes are related to enzyme activity. Many previous studies have related soil enzyme activities with their respective enzyme-encoding genes [60,61,62]. The significant relationship of cellobiohydrolase activity to soil pH (*p* = 0.037) and the abundance of *cbh*I gene (*p* < 0.001) (Figure 4) also supported these relationships. The increase in SOC mineralization after forest conversion can be regulated by increased cellobiohydrolase activities [31,32,34].

Many studies have demonstrated that converting natural forests to intensively managed plantations decrease SOC storage [13,63,64]. However, forest conversion in our study did not significantly change SOC storage in the surface layer, which may be related to the nature of the extensive management in MBP. The litter from above- and below-ground are important sources of SOC accumulation [65], and the quality and quantity of forest litter input are responsible for the SOC concentration and storage [52,66]. In the current study, the vegetation was gradually restored and the understory vegetation was also retained following the long-term extensive management after forest conversion to MBP, which subsequently maintained the abundant plant C input to the soil, resulting in the lack of significant changes in the SOC concentration. Furthermore, lack of ploughing in MBP did not affect soil BD between EBF and MBP. The lack of forest conversion effect on SOC concentration and BD resulted in the lack of change in SOC storage. In spite of this, if the higher SOC mineralization rate after forest conversion is not abated, it could decrease the SOC storage in MBP if the input of litter decreases.

## 5. Conclusions

Our study provides new insights into the effect of converting natural forests to extensively managed Moso bamboo plantations on SOC mineralization rate, which was linked to activities of C-degrading enzymes. The forest conversion increased activities of cellobiohydrolase and phenol oxidase in the extensively managed Moso bamboo plantation, which could be explained by a higher soil pH, *cbh*I gene abundance, and MBC concentration. The increased activities of C-degrading enzymes, especially that of cellobiohydrolase, significantly increased the SOC mineralization rate. The lack of change in SOC storage following the forest conversion suggests that the rate of organic C input in the extensively managed bamboo forest was greater than the rate of SOC mineralization. The enhanced SOC mineralization after forest conversion does suggest that the risk of SOC loss and reduction in SOC storage may increase in the event that the rate of organic C input in the form of above- and below-ground litterfall falls below the rate of organic C mineralization. Before making decisions on land-use conversion, the impact of such conversions on the C cycle in the ecosystem needs to be carefully studied to ensure that no negative effect on SOC sequestration is caused by the conversion, otherwise such conversions should be avoided as much as possible.

## Figures and Tables

**Table 1:** Effects of conversion of evergreen broadleaf forests (EBF) to extensively managed Moso bamboo plantations (MBP) on selected soil properties (n = 3).

Soil Property		Sampling Time	EBF	MBP	*p*-Value (Paired *t*-Tests)
Bulk density (g cm^−3^)		July	1.52 ± 0.06	1.23 ± 0.11	0.227
	Clay (%)	July	32.46 ± 1.30	31.89 ± 0.17	0.668
Texture	Silt (%)		44.44 ± 0.43	45.06 ± 2.09	0.783
	Sand (%)		23.10 ± 1.44	23.05 ± 1.92	0.988
Moisture content (%)		July	22.45 ± 2.67	24.18 ± 4.34	0.604
		October	12.59 ± 0.83	14.57 ± 0.20	0.183
		Mean	17.52 ± 2.54	19.37 ± 2.90	0.225
pH (H_2_O)		July	4.33 ± 0.03	4.64 ± 0.07	0.023
		October	4.17 ± 0.04	4.55 ± 0.03	0.003
		Mean	4.25 ± 0.04	4.60 ± 0.04	<0.001
Soil organic carbon (SOC) (mg kg^−1^)		July	21.12 ± 1.39	23.61 ± 2.01	0.209
		October	20.64 ± 7.01	22.84 ± 4.76	0.445
		Mean	20.88 ± 3.20	23.22 ± 2.32	0.110
Total nitrogen (mg kg^−1^)		July	1.04 ± 0.04	1.67 ± 0.26	0.151
		October	1.69 ± 0.13	1.93 ± 0.32	0.603
		Mean	1.37 ± 0.16	1.80 ± 0.19	0.119
C/N		July	20.45 ± 2.14	14.74 ± 2.13	0.135
		October	12.93 ± 5.30	11.86 ± 1.34	0.838
		Mean	16.69 ± 3.06	13.30 ± 1.30	0.240
SOC storage (Mg ha^−1^)		July	64.31 ± 6.27	58.27 ± 8.34	0.634
		October	64.38 ± 4.62	54.00 ± 5.66	0.639
		Mean	64.34 ± 3.59	56.13 ± 4.61	0.442

## Data Availability

Not applicable.
